# A first-in-human phase 1 study of cavrotolimod, a TLR9 agonist spherical nucleic acid, in healthy participants: Evidence of immune activation

**DOI:** 10.3389/fimmu.2022.1073777

**Published:** 2022-12-13

**Authors:** Weston L. Daniel, Ulrike Lorch, Scott Mix, Alice S. Bexon

**Affiliations:** ^1^ Research and Development, Exicure, Inc., Chicago, IL, United States; ^2^ Clinical Research, Richmond Pharmacology, London, United Kingdom; ^3^ Clinical Research, Bexon Clinical Consulting, Upper Montclair, NJ, United States

**Keywords:** immunotherapy, cytokines, immunomodulation, Toll-like receptor, clinical trial, cavrotolimod, AST-008, spherical nucleic acid

## Abstract

**Introduction:**

Tumor immunotherapy is designed to control malignancies through the host immune response but requires circumventing tumor-dysregulated immunomodulation through immunostimulation, relieving immunorepression, or a combination of both approaches. Here we designed and characterized cavrotolimod (formerly AST-008), an immunostimulatory spherical nucleic acid (SNA) compound targeting Toll-like receptor 9 (TLR9). We assessed the safety and pharmacodynamic (PD) properties of cavrotolimod in healthy participants in a first-in-human Phase 1 study under protocol AST-008-101 (NCT03086278; https://clinicaltrials.gov/ct2/show/NCT03086278).

**Methods:**

Healthy participants aged 18 to 40 years were enrolled to evaluate four dose levels of cavrotolimod across four cohorts. Each cohort included four participants, and all received a single subcutaneous dose of cavrotolimod. The dose levels were 5, 10, 12.5 and 18.8 µg/kg.

**Results and discussion:**

Cavrotolimod was well tolerated and elicited no serious adverse events or dose limiting toxicities at the doses tested. The results demonstrated that cavrotolimod is a potent innate immune activator, specifically stimulating Th1-type immune responses, and exhibits PD properties that may result in anti-tumor effects in patients with cancer. This study suggests that cavrotolimod is a promising clinical immunotherapy agent.

## Introduction

The immune system is the body’s defense mechanism against foreign and infectious agents, such as bacteria and viruses. In addition to combating these dangers, the immune system can also fight diseases like cancer. Tumor immunotherapy uses the immune system to recognize and kill cancerous cells. However, tumors can use immune-checkpoint pathways as a major mechanism of immune resistance, particularly against T cells that are specific for tumor antigens. These checkpoint pathways can prevent a latent immune response from acting on the tumor.

Tumor immunotherapy has been revolutionized by checkpoint inhibitors (CPIs) (1) targeting immune checkpoints such as the programmed cell death 1 protein (PD-1)/PD ligand (L)1 axis and cytotoxic T lymphocyte antigen 4 (CTLA-4). Agents targeting both pathways have been approved for use in many cancer types. CPI therapy reduces the inhibition of anti-tumor immunity, allowing the immune system to act more strongly on cancers, resulting in tumor regression and long-term responses in 10–50% of patients (2, 3). Despite these successes, there are two key opportunities for improvement. First, because only a minority of patients treated with CPIs benefit clinically, new approaches including combination therapies increasing the proportion of responders are needed. Second, because there are limited treatment options after disease progression on CPIs, additional therapeutic options are needed in that setting. We hypothesized that combining a potent TLR9 agonist (an immune stimulant) with CPIs (which support immune response expansion) would be a logical approach for capitalizing on the complementary nature of these two therapeutic mechanisms to begin the self-promoting process of immune-mediated tumor cell death, called the cancer-immunity cycle (4), and address the two key therapeutic opportunities outlined above. TLR9 is a pattern recognition receptor (PRR) that recognizes nucleic acids containing unmethylated cytosine-phosphate-guanosine (CpG) dinucleotides present in specific sequence contexts, referred to as CpG motifs (5–9), resulting in T-helper 1 (Th1)-type innate and adaptive immune responses (10, 11). Innate immunity is a rapid response that protects the body during the time required for an adaptive immune response to develop against a given threat, which is usually a few days to weeks. Antigen-presenting cells, including dendritic cells, macrophages, and B cells, play a critical role in the innate immune response by releasing protein signals called cytokines that induce inflammation to fight invading pathogens or foreign bodies. In addition, they direct T cells, which then coordinate the longer-term adaptive immune response, conferring long-term pathogen-specific immunity to the host.

TLR9 agonists have been extensively evaluated preclinically (12–14) and clinically as agents for treating cancers, asthma and allergies, infectious diseases, and as vaccine adjuvants (15, 16). Despite good safety profiles, conventional TLR9 agonists, such as SD-101 and tilsotolimod, have resulted in varied anti-tumor responses as monotherapies or when combined with other anticancer agents following systemic administration (16–18), suggesting a need for more potent TLR9 agonists and better combination approaches. A TLR9 agonist packaged within a virus-like particle called vidutolimod showed substantial clinical activity when administered in combination with pembrolizumab in advanced and metastatic melanoma patients who had previously progressed on anti-PD-1 therapy alone (18), but the complexity of preparing the virus capsid and oligonucleotide components, and assembling the virus-like particle structure, may limit its widespread application.

**Figure 1:**
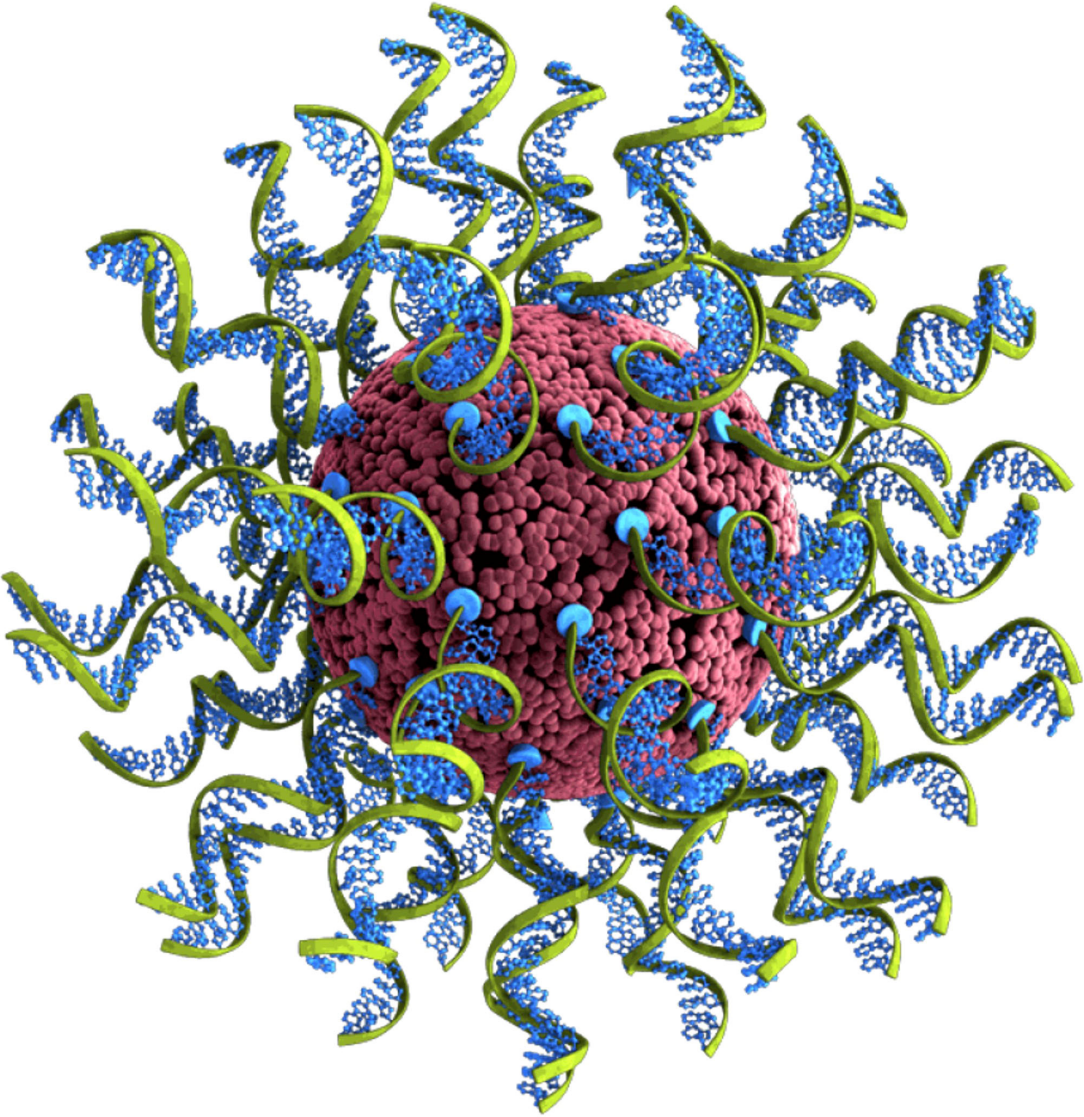
Depiction of the cavrotolimod spherical nucleic acid structure.

Spherical nucleic acids (SNAs) are an emerging category of therapeutic agents, showing promise for treating a wide range of diseases and debilitating conditions, spanning glioblastoma multiforme (19, 20), psoriasis (21), diabetic wound healing (22), and nonalcoholic steatohepatitis (23), among others. As illustrated in **Figure 1**, SNA structures consist of densely packed and radially oriented oligonucleotides around a spherical nanoparticle core (24–27). Since these structures enter cells to a greater extent compared to their conventional oligonucleotide analogues (i.e., unformulated oligonucleotides) and do so *via* endosomal pathways (28), they are ideal for interacting with targets that reside in endosomes, such as TLR9 (28–30). Moreover, since the oligonucleotides on the surface of SNAs are projected in a way that supports facile TLR9 binding (i.e., the oligonucleotides are not sequestered within the nanoparticle), such structures are more potent stimulatory agents compared to unformulated oligonucleotides (23).

We developed cavrotolimod, an SNA modified with type B CpG oligonucleotides designed to agonize TLR9 and elicit immune responses useful in oncology applications, and assessed its PD and safety in a Phase 1 study. The results indicated that cavrotolimod produces AEs and a PD response consistent with immune activation, suggesting that cavrotolimod may synergize effectively with anti-PD-1/PD-L1 antibodies. This study, along with data from nonclinical efficacy studies showing tumor regression in mice (31), supported the clinical evaluation of cavrotolimod in combination with CPIs in patients with advanced cancer.

## Materials and methods

### Study design

This was an open label Phase 1 study which was designed to assess the safety, tolerability, PK and PD of single ascending doses of subcutaneously (SC) administered cavrotolimod in healthy participants. The objectives and endpoints for this study were: (I) to evaluate the safety and tolerability of cavrotolimod; (II) to recommend a dose and regimen for further development; (III) to determine the PK of cavrotolimod in plasma and urine; (IV) to determine the PD of cavrotolimod; and (V) to determine the effect of cavrotolimod on the corrected QT (QTc) interval. The study was conducted at a single clinical study unit in the United Kingdom (UK) and enrolled four cohorts of four participants to receive single ascending doses of cavrotolimod. Participants were assigned into sequential cohorts that received cavrotolimod doses of 5, 10, 12.5 or 18.8 µg/kg. These doses were selected by applying a cautious multiple between these human doses and the no observed adverse event level of cavrotolimod in monkey toxicology studies. In addition, results from a study of TLR9 agonist PF-3512676 in healthy participant were considered (32). No participants received placebo. The study was approved and had oversight by the South Central – Berkshire B Research ethics committee and the Medicines Healthcare Products Regulatory Agency, and was conducted according to the Declaration of Helsinki and Good Clinical Practices.

**Figure 2:**
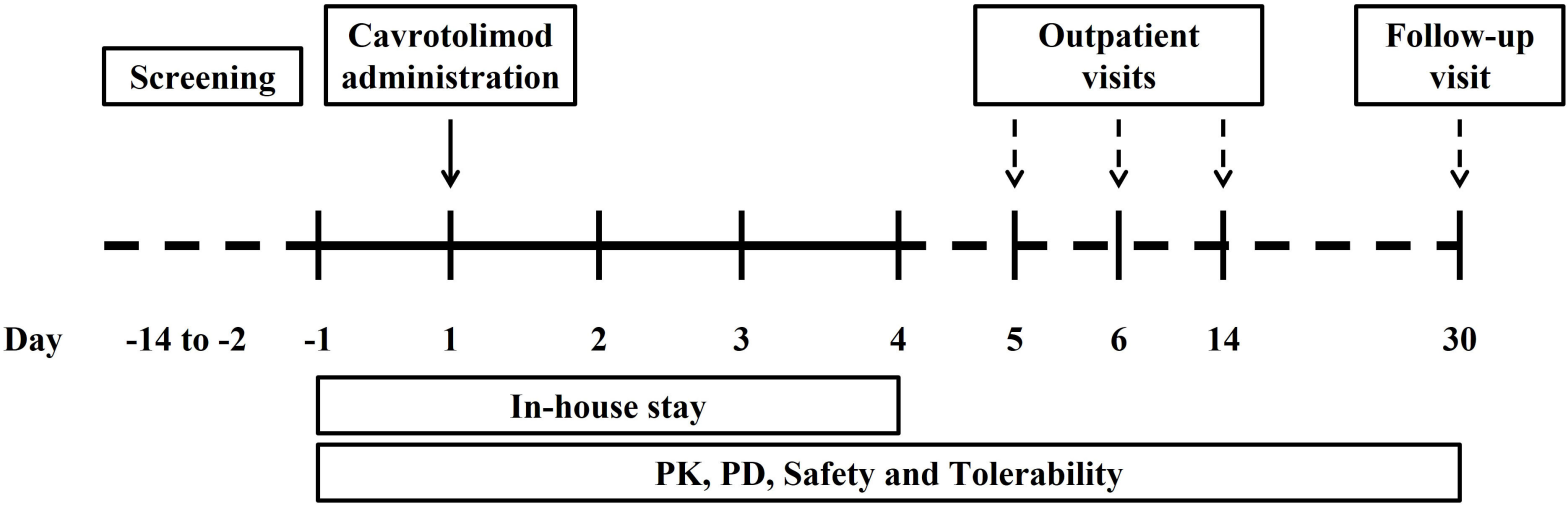
Flow chart for the phase 1 study.

The main criteria for admission were healthy females or males aged between 18 and 40 years with a body mass index between 18.0 and 25.0 kg/m^2^. Participants must have agreed to use effective methods of contraception, if applicable. The main exclusion criteria were any history of cancer and autoimmune or antibody-mediated diseases or any other significant disease or disorder which, in the opinion of the investigator, may have either put the participant at risk because of participation in the study, may have influenced the result of the study or the participant’s ability to participate in the study. Participants were screened within 13 days prior to entering the unit on Day -1. Each participant received verbal and written information followed by signing of the Informed Consent Form prior to any screening procedures taking place. Participants were randomized to one dose cohort, admitted to the study unit on Day -1, dosed on Day 1 and discharged on Day 4. All participants attended the unit for outpatient visits on Days 5, 6 and 14 and a follow-up visit on Day 30 (**Figure 2**).

A mandatory sentinel dosing strategy was used in each cohort, whereby participants were dosed with intervals of 24-96 hours (if safety and tolerability was acceptable) following the prior participant. The Investigator made a judgement whether administration of cavrotolimod to the remaining participants in the cohort could continue based on the clinical safety data available at the time and the protocol toxicity rules. Dose escalation to the subsequent cohort/dose could only occur after satisfactory review of all safety, tolerability, PK and PD data by the Safety Review Committee (SRC) in accordance with the protocol, rules for dose escalation/progression, and toxicity.

### Drug supply and administration

A single batch of cavrotolimod was prepared under current Good Manufacturing Practices and released by a Qualified Person to support this study. The product was supplied in single use glass vials as a sterile, pH-controlled isotonic solution. To achieve the appropriate doses for each cohort in the study, the pharmacist prepared dilutions of the product in simple saline prior to administration. Cavrotolimod was administered as a SC injection into the abdomen using a 25-gauge, one inch needle.

### Safety assessments

Safety and tolerability assessments included AE recording, general health and concomitant medication assessments, and hematology/biochemistry/coagulation parameters during screening, before cavrotolimod dosing, daily during the admission to the study unit, and during the outpatient and follow up visits. At screening and while admitted to the unit, participants were interrogated with continuous 12-lead telemetry starting 1 hour pre-dose through 48 hours post-dose. Further, a Holter electrocardiogram (ECG) was performed during screening to exclude any pre-existing ECG abnormalities. Urinalysis was performed at screening, pre-dose, and Days 3, 14, and 30. Chemokine/cytokine sampling was performed pre- and post-dose during admission, as well as during the outpatient visits and follow up. Lymphocyte sampling was performed pre-dose through Day 3 for the 5 and 10 µg/kg dose groups, and from pre-dose to Day 5 for the 12.5 and 18.8 µg/kg dose groups. PK blood sampling was performed pre- and post-dose through Day 6. PK urine sampling was performed pre-dose and post-dose through Day 2.

### Toxicity rules and management algorithm

There were two sets of toxicity rules used in this study. One applied to flu-like symptoms and cytokine release syndrome (CRS), while the second concerned all other adverse events. The rationale for this approach was twofold. First, several constitutional symptoms are present in both flu-like symptoms and CRS, but CRS is a more significant AE, and second, flu-like symptoms were expected after cavrotolimod administration. Therefore, special attention was paid to the toxicity definitions and management of potential CRS reactions.

A CRS management approach based on the literature was used during the first cohort of the study, but for the final three cohorts, a modified algorithm was developed and applied because the approach outlined in the literature was designed for patients who would most likely be in a hospital setting (33). Modifications to the algorithm were therefore made to reflect its application for healthy participants in a non-hospital setting.

**Figure 3:**
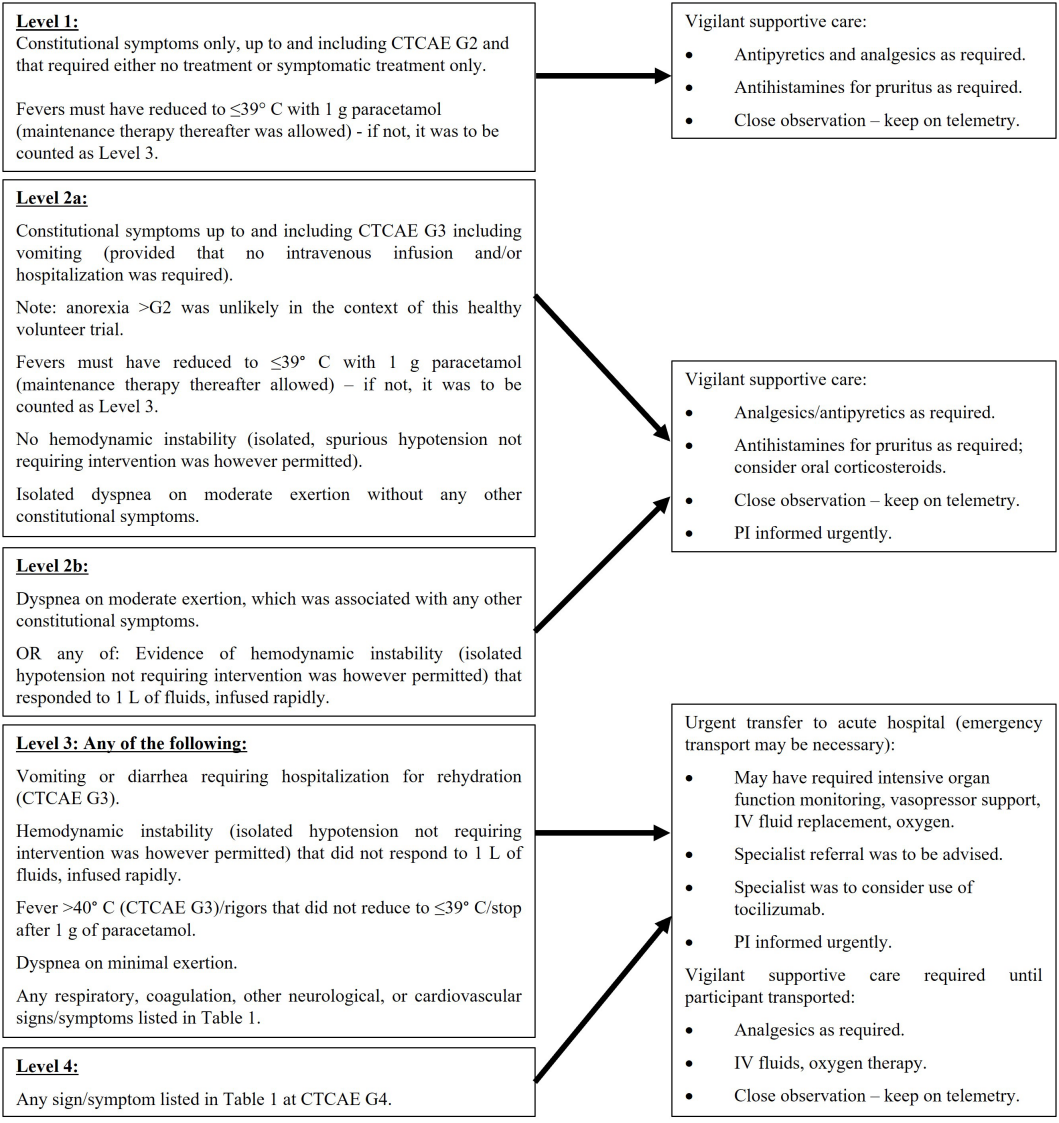
Definitions of severity levels and management algorithm for flu-like symptoms/CRS.

The constitutional symptoms and signs of CRS and flu-like symptoms at mild to moderate severity are similar. **Table 1** lists the signs and symptoms of CRS and those in bold overlap with the presentation of flu-like symptoms. The management of any AE comprised of the signs or symptoms shown in **Table 1** was therefore based on the severity of the overall clinical picture and is illustrated in the algorithm in **Figure 3**.

**Table 1:** Clinical signs and symptoms associated with CRS. Bold indicates signs and symptoms also seen in flu-like symptoms.

Organ system	Symptoms
Constitutional	Fever ± rigors, malaise, fatigue, anorexia, myalgia, arthralgia, nausea, vomiting, and headaches. Additionally, pruritus and dizziness were observed with compounds similar to cavrotolimod and for the purposes of this management algorithm will also be regarded as constitutional, unless another cause for these symptoms is identified.
Skin	Rash.
Gastrointestinal	Nausea, vomiting, diarrhea.
Respiratory	Tachypnoea, hypoxemia.
Cardiovascular	Tachycardia, widened pulse pressure, hypotension, increased cardiac output (early), potentially diminished cardiac output (late).
Coagulation	Elevated D-dimer, hypofibrinogenemia ± bleeding.
Renal	Azotemia.
Hepatic	Transaminitis, hyperbilirubinemia.
Neurologic	Headache, mental status changes, confusion, delirium, word finding difficulty or frank aphasia, hallucinations, tremor, dysmetria, altered gait, seizures.

The toxicity rules that governed study progression of individual participants, cohorts and the overall study in the event of these AEs were based on the same objective severity scale used in the management algorithm and are shown in **Table 2**. Based on the desired pharmacology of stimulating a Th1-type immune response, TLR9 agonists are known to cause a range of local and systemic effects that are not necessarily considered adverse. In general, in healthy participant studies of TLR9 agonists administered SC, participants typically experience at least one of the following AEs: injection site induration, erythema, swelling and tenderness, headache, chills, pyrexia/fever, myalgia, arthralgia (constellation of the 5 latter symptoms often described as “flu-like symptoms”).

**Table 2:** Toxicity rules for CRS and Flu-like symptoms.

Level (see Figure 3 for explanation on levels)	DECISION 1	DECISION 2	DECISION 3
	Individuals:	Continuation within a dosing regimen:	Study progression:
**Level 1** (mild to moderate, not serious)	Continue as per protocol.	Continue as per protocol.	Continue as per protocol.
**Level 2a** (Severe, not serious)	Cavrotolimod administration was to be discontinued.	1 participant: Continue as per protocol. ≥2 participants: Dosing of the remainder of the dosing regimen suspended. Continuation and extension required substantial amendment.	1 participant: Continue as per protocol. ≥2 participants: Dose escalation and progression to study parts with an equal or higher dose suspended. Dosing regimens on lower dose levels can continue. Progression to successive cohorts or study parts was permitted only with doses below this current level (at which this toxicity was observed). Escalation or progression to study parts with an equal or higher dose requires substantial amendment.
**Level 2b** (Severe, not serious)	Cavrotolimod administration was to be discontinued.	≥1 participant: Dosing of the remainder of the dosing regimen suspended. Continuation and extension required substantial amendment.	≥1 participant: Dose escalation and progression to study parts with an equal or higher dose suspended. Dosing regimens on lower dose levels can continue. Progression to successive cohorts or study parts was permitted only with doses below this current level (at which this toxicity was observed). Escalation or progression to study parts with an equal or higher dose required substantial amendment.
**Level 3** (Severe, serious AE)	Cavrotolimod administration was to be discontinued.		
**Level 4** (Life-threatening, serious AE)	Cavrotolimod administration was to be discontinued.	≥1 participants: Dosing of the remainder of the dosing regimen suspended; Continuation and extension requires substantial amendment.	Study suspended (i.e., this dosing regimen AND all ongoing dosing regimens including those at lower doses, and upcoming dosing regimens, were to be immediately suspended). Continuation of the study required a substantial amendment.

### Pharmacokinetic assessments

Venous blood samples were collected to assess the concentration of cavrotolimod in plasma (K_2_EDTA). Blood was collected pre- and post-dose through Day 6. Urine samples for cavrotolimod concentration measurements were taken from the total urine sample provided pre-dose and post-dose through Day 2. Cavrotolimod concentrations were assessed in plasma and urine with validated assays. Non-compartmental analysis was used for calculation of plasma PK parameters. More information about the assays and PK analysis are provided in the Supporting Information.

### Pharmacodynamic assessments

Blood samples were taken for the evaluation of the mechanism of action-related cytokines and chemokine markers and immune cell activation. Chemokine and cytokine sampling was performed pre- and post-dose during admission, as well as during the outpatient visits and follow up. Concentrations of cytokines and chemokines were assessed in plasma with a Randox Evidence Investigator or ELISA. The cytokine and chemokines measured were interferon (IFN)α, IFNγ, interleukin (IL)-10, IL-12 p40, IL-1β, IL-1 receptor (R)a, IL-2, IL-6, IL-8, interferon gamma-induced protein (IP)-10, monocyte chemoattractant protein (MCP)-1, and tumor necrosis factor (TNF)α.

Lymphocyte sampling was performed pre-dose through Day 3 for the 5 and 10 µg/kg dose groups, and from pre-dose to Day 5 for the 12.5 and 18.8 µg/kg dose groups. Differential lymphocyte count was performed on a Beckman Coulter Navios flow cytometer to measure abundance of B-cells, T-cells, natural killer (NK) cells, monocytes, macrophages, and plasmacytoid dendritic cells. NK and T-cells were identified as CD3-/CD16+/CD19-/CD56+ and CD3+/CD19-/CD45+, respectively. Monocytes were detected as CD14+/CD45+. CD69 was used as the activation marker for these three cell types.

Cytokines, chemokines and flow cytometry analyses were performed by The Doctors’ Laboratory (London, UK).

### Statistical analysis

A blinded data review meeting was held prior to database lock and completion of the final analyses. AEs, vital signs, ECG parameters and clinical laboratory data were listed and summarized using descriptive statistics. The number and percent of participants who had any AEs were summarized for each dose. All AEs were listed by using system organ class and preferred term assigned to the event using Medical Dictionary for Regulatory Activities (MedDRA). Furthermore, these events were summarized by the maximum intensity and the number of participants who had drug-related AEs. Any serious adverse events (SAEs) and/or AEs that led to withdrawal would have been listed had they occurred.

The statistical analysis of the PD variables consisted of descriptive statistics, listings and graphs of absolute values and fold change from pre- to post-dose time points, and of comparison of pre- to post-dose values by paired t-tests.

## Results

### Participant characteristics

Cavrotolimod was evaluated in a first-in-human Phase 1 study (34), which was conducted at a single clinical study unit in the UK. The safety, tolerability, PK and PD of single ascending doses of cavrotolimod were studied in healthy participants. Four dose levels of cavrotolimod (5, 10, 12.5, and 18.8 µg/kg) were evaluated in four cohorts. Each cohort included four participants, and all received a single dose of cavrotolimod.

A total of 16 healthy female and male participants were dosed, with the highest single dose of cavrotolimod being 1.4 mg. Among the 16 participants, 13 were male and 3 were female (**Table 3**). The mean age was 23.9 years (range from 18-37 years), and the mean weight was 69.6 kg (ranging from 54.4 kg to 84.1 kg) at screening. The participants had a mean height of 176.0 cm (ranging from 158 cm to 192 cm). Participants had a mean BMI of 22.4 kg/m^2^ (ranging from 19 kg/m^2^ to 25 kg/m^2^ at screening. A quarter (25%) of the participants who received cavrotolimod were black, while 56.3% were white and 18.8% were of ‘other’ race. The participants enrolled into the trial were all included in the safety, PK and PD analyses. There were no dropouts.

**Table 3:** Demographic data of healthy participants enrolled in AST-008-101.

		5 µg/kg	10 µg/kg	12.5 µg/kg	18.8 µg/kg	Overall
Demographic Parameter	Statistic	(N=4)	(N=4)	(N=4)	(N=4)	(N=16)
Age* [years]	n	4	4	4	4	16
	Mean	21.8	22.5	28.8	22.5	23.9
	SD	2.6	3.7	5.6	1.3	4.4
	Minimum	18	20	25	21	18
	Median	22.5	21.0	26.5	22.5	23.0
	Maximum	24	28	37	24	37
**Gender**	Female, n (%)	1 (25.0)	0	0	2 (50.0)	3 (18.8)
	Male, n (%)	3 (75.0)	4 (100.0)	4 (100.0)	2 (50.0)	13 (81.3)
**Race**	Black or African American, n (%)	1 (25.0)	0	1 (25.0)	2 (50.0)	4 (25.0)
	Other, n (%)	0	1 (25.0)	1 (25.0)	1 (25.0)	3 (18.8)
	White, n (%)	3 (75.0)	3 (75.0)	2 (50.0)	1 (25.0)	9 (56.3)
Height** [cm]	n	4	4	4	4	16
	Mean	175.3	185	176.3	167.5	176
	SD	11.6	4.8	5.3	12.3	10.4
	Minimum	158	181	172	158	158
	Median	180.0	183.5	174.5	163.5	180
	Maximum	183	192	184	185	192
Weight** [kg]	n	4	4	4	4	16
	Mean	68.7	77.95	68.58	63.13	69.59
	SD	10.29	7.26	10.60	6.33	9.62
	Minimum	54.4	67.6	55.7	59.6	54.4
	Median	70.75	80.05	69.4	60.15	70.75
	Maximum	78.9	84.1	79.8	72.6	84.1
**Body Mass Index [kg/m²]**	n	4	4	4	4	16
	Mean	22.3	22.8	22.0	22.6	22.4
	SD	0.96	1.82	2.57	1.45	1.64
	Minimum	21	20	19	21	19
	Median	22.1	23.4	22.4	22.5	22.6
	Maximum	24	24	25	24	25

N, total number of participants in cohort; n, number of participants for a given statistic; SD, standard deviation. * Age is calculated at the date of informed consent. ** Height, weight, and body mass index are given at screening.

### Safety and tolerability assessments

Safety and tolerability assessments included AE recording, general health and concomitant medication assessments, hematology, biochemistry and coagulation parameters measured during screening, before cavrotolimod dosing, daily during the admission to the study unit, and during the outpatient and follow up visits.

All participants at screening, on Day -1, and Day 1 met all inclusion criteria, and none violated any of the exclusion criteria. Two participants reported previous medical history. One participant who received 5 µg/kg of cavrotolimod reported depression and one participant who received 10 µg/kg reported seasonal allergy and depression. Prior and concomitant medication was reported in nine participants. Prior and concomitant medications related to AEs included norfloxacin for urinary tract infections, Strepsils^®^ for sore throat, ibuprofen for toothache and flu-like symptoms, lidocaine for toothache, paracetamol (acetaminophen) for headache, hot flush and pyrexia. Prior and concomitant medication use not related to AEs included loratadine for a runny nose and omega-3 fatty acids for health and well-being.

In the trial, no SAEs or dose limiting toxicities were observed. All 16 participants receiving cavrotolimod experienced at least one drug-related AE, all of which were grade (G) 1 in severity according to the Common Terminology Criteria for Adverse Events (CTCAE) version 4.03 (**Table 4**). The AEs observed were as expected given the mechanism of action and from other TLR9 agonist clinical trials in healthy participants (32, 35) and in diseased populations (16, 36). The frequency of AEs was similar in the lowest three dose groups but increased in the highest dose group. The most common AEs, in descending order of frequency, were pyrexia, headache, influenza-like illness (i.e., flu-like symptoms), dizziness and myalgia. All 16 participants experienced mild injection site reactions (i.e., redness, tenderness) that were not considered adverse, and these reactions resolved on their own. Lymphadenopathy was observed in 12 participants but was considered adverse in only one participant. Across all timepoints, the highest grading was ‘palpable, normal to mild enlargement’. The inguinal, femoral, and iliac lymph nodes were most often affected, due to the lower right quadrant dosing of cavrotolimod.

**Table 4:** Drug-related adverse events (all Grade 1) observed in AST-008-101.

Preferred Term (MedDRA 21.0)	5 µg/kg	10 µg/kg	12.5 µg/kg	18.8 µg/kg	Overall
	(N=4)	(N=4)	(N=4)	(N=4)	(N=16)
	n (%)	n (%)	n (%)	n (%)	n (%)
Pyrexia	–	–	2 (50.0)	4 (100.0)	6 (37.5)
Influenza like illness	–	3 (75.0)	–	1 (25.0)	4 (25.0)
Headache	1 (25.0)		1 (25.0)	2 (50.0)	4 (25.0)
Myalgia	–	–	–	2 (50.0)	2 (12.5)
Dizziness	–	–	1 (25.0)	1 (25.0)	2 (12.5)
Lymphadenopathy	–	1 (25.0)	–	–	1 (6.3)
Eye pain	1 (25.0)	–	–	–	1 (6.3)
Decreased appetite	–	–	–	1 (25.0)	1 (6.3)
Back pain	–	–	1 (25.0)	–	1 (6.3)
Muscle twitching	1 (25.0)	–	–	–	1 (6.3)
Hyperesthesia	–	–	–	1 (25.0)	1 (6.3)
Cough	–	–	–	1 (25.0)	1 (6.3)
Flushing	–	–	1 (25.0)	–	1 (6.3)

N, total number of participants in cohort; n, number of participants for a given statistic.

Clinical biochemistry and hematology results revealed C-reactive protein increases, neutropenia, and lymphopenia, but these changes were not considered adverse due to their short-lived and asymptomatic nature. Neutropenia and lymphopenia are consistent with the pharmacology of cavrotolimod as they were likely due to the transient margination, or exit, of those cells from the bloodstream.

No clinically significant abnormalities in ECG intervals or morphology were observed. Although minor abnormalities of ECG intervals were reported at all doses, most were either not considered to be changes from the subject’s pre-dose baselines or were related to spurious results or anomalies and were not reproducible at subsequent time points. The paired PK/QTc analysis was not conducted because of a lack of change in QTc parameters.

No CRS/flu-like symptoms toxicity rules above Level 1 were met over the course of the study.

### Pharmacokinetic assessments

The PK of cavrotolimod was evaluated after single SC administration in healthy adult participants. Eighteen blood samples were collected starting at the pre-dose baseline through 120 hours after the cavrotolimod dose. The samples were used to measure the concentration of the drug in the participant plasma with a plate-based method, utilizing complementary capture and detection probes, and an ECL readout. There were only a few measurable plasma concentrations per participant. Due to this and the small number of participants, these PK results should be interpreted with caution. PK parameters, including AUC_0-24hr_, AUC_0-tlast_, C_max_, the time of maximum plasma concentration (T_max_) and plasma half-life (t_1/2_) were calculated and are presented by dose in **Table 5**. The geometric mean AUC_0-24hr_, AUC_0-tlast_ and C_max_ values were similar for the 5 and 10 µg/kg dose groups, and increased in a dose proportional manner in the higher dose groups.

**Table 5:** PK parameters calculated by cavrotolimod dose.

	Cavrotolimod dose			
PK Parameter	5 µg/kg	10 µg/kg	12.5 µg/kg	18.8 µg/kg
AUC_0-24hr_ (ng × hr/mL)	2.302	2.084	4.762	8.170
AUC_0-tlast_ (ng × hr/mL)	1.992	1.817	4.241	8.565
C_max_ (ng/mL)	0.74009	0.73726	1.3336	1.8234
T_max_ (hr)	2.52	2.00	2.00	2.04
T_1/2_ (hr)	NC	NC	3.881	2.370
CL (L/h/kg)	NC	NC	1.133	2.266
Vz (L/kg)	NC	NC	6.344	7.748

CL, clearance; hr, hour; kg, kilogram; L, liter; mL, milliliter; NC, not calculated; ng, nanogram; Vz, volume of distribution during the terminal phase.

The median T_max_ ranged from 2.00 to 2.52 hours over the dose range of 5 µg/kg to 18.8 µg/kg, peaking earlier than the PD effects, which apexed at 24 to 72 hours, depending on the cytokine. Geometric mean t_1/2_ values were 3.881 and 2.370 hours following 12.5 and 18.8 µg/kg administration, while geometric mean apparent clearance (CL) and volume (Vz) were 1.133 L/h/kg and 6.344 L/kg for the 12.5 µg/kg dose level, and 2.266 L/h/kg and 7.748 L/kg for the 18.8 µg/kg dose level, respectively. Inter-participant variability for AUC_0-24h_, AUC_0-tlast_, C_max_ were generally low to moderate, except for the 12.5 µg/kg dose level which was generally high.

Cavrotolimod was not detected in any urine samples, so PK parameter analysis was not conducted on those samples.

### Pharmacodynamic assessments

The PD effects of cavrotolimod were assessed through the measurement of cytokine and chemokine concentrations and lymphocyte changes. Blood was drawn from the participants before and after dosing with cavrotolimod to measure the concentration of a set of cytokines and chemokines in the participant plasma as a function of time. Blood samples were also used for fresh flow cytometry to assess immune cell activation as a percent of the cells detected by flow cytometry.

The cytokine and chemokine markers measured in the study were IL-12 p40, IL-1RA, IP-10, IL-6, MCP-1, TNFα, IFNα, IFNγ, IL-10, IL-1β, IL-2, and IL-8. Approximately 18 blood samples were taken from the participants during a period starting at the pre-dose time point out to 30 days post-dose in each participant. These markers were selected to assess the magnitude and character of the immune response provoked by cavrotolimod. **Table 6** shows the peak baseline-subtracted cytokine change with cavrotolimod as a function of dose.

**Table 6:** Peak cytokine response by cohort following cavrotolimod administration.

	Peak Cytokine Response (expressed in pg/mL)								
	5 µg/kg		10 µg/kg		12.5 µg/kg		18.8 µg/kg		
Cytokine	mean ± SD	p value	mean ± SD	p value	mean ± SD	p value	mean ± SD	p value
IFNα	11 ± 27	0.4617	7 ± 4	0.0505	4 ± 6	0.2900	1 ± 6	0.8156
IFNγ	2 ± 0	0.0033	12 ± 8	0.0577	3 ± 3	0.1156	5 ± 2	0.0207
IL-1β	1 ± 1	0.3910	0 ± 1	0.3910	0 ± 1	0.3910	2 ± 3	0.2380
IL-1RA	857 ± 288	0.0094	1763 ± 1024	0.0412	1073 ± 576	0.0337	1519 ± 500	0.0089
IL-2	1 ± 2	0.1828	3 ± 4	0.1818	3 ± 2	0.0822	0 ± 3	0.7915
IL-6	20 ± 16	0.0772	123 ± 48	0.0147	64 ± 81	0.2121	66 ± 99	0.2773
IL-8	4 ± 7	0.3557	5 ± 5	0.1512	22 ± 42	0.3634	15 ± 15	0.1457
IL-10	6 ± 9	0.2586	7 ± 9	0.2030	1 ± 1	0.0524	1 ± 1	0.2188
IL-12 p40	99 ± 57	0.0402	192 ± 149	0.0826	266 ± 30	0.0004	263 ± 157	0.0442
IP-10	277 ± 144	0.0309	721 ± 236	0.0088	687 ± 241	0.0107	1007 ± 342	0.0098
MCP-1	276 ± 115	0.0173	753 ± 613	0.0912	303 ± 84	0.0055	420 ± 188	0.0209
TNFα	2 ± 1	0.0180	17 ± 27	0.3037	2 ± 3	0.3233	46 ± 89	0.3788

Peak cytokine response (mean ± standard deviation) and p values (comparison of pre- to post-dose values by paired t-tests) by cohort as a function of dose (baseline subtracted). pg/mL, picogram per milliliter; SD, standard deviation.

Cavrotolimod elicited a broad Th1-type immune response in an approximately dose-proportional manner. IFNγ, IL-12 p40, IL-1RA, IL-6, IP-10, and MCP-1 were consistently induced across all doses. IFNγ increases were small, but statistically significant, at 20 hours post-dose in all four cohorts. Notably, IP-10 was robustly induced compared to pre-dose levels reaching significance (p<0.05) in all four cohorts between 12- and 24-hours post-dose, providing a second indication that IFNγ was elicited, although the latter was not detected at the same magnitude. IFN-γ expression induces PD-L1, a marker positively associated with anti-PD-1 antibody activity (37). TNF-α was also induced, although not consistently or in a dose-proportionate manner. Two participants showed a substantial increase in TNF-α, to 58 and 179 pg/mL above baseline, at the 10 and 18.8 µg/kg dose levels, respectively, while the other participants in the study did not express meaningful levels of the cytokine.

**Figure 4:**
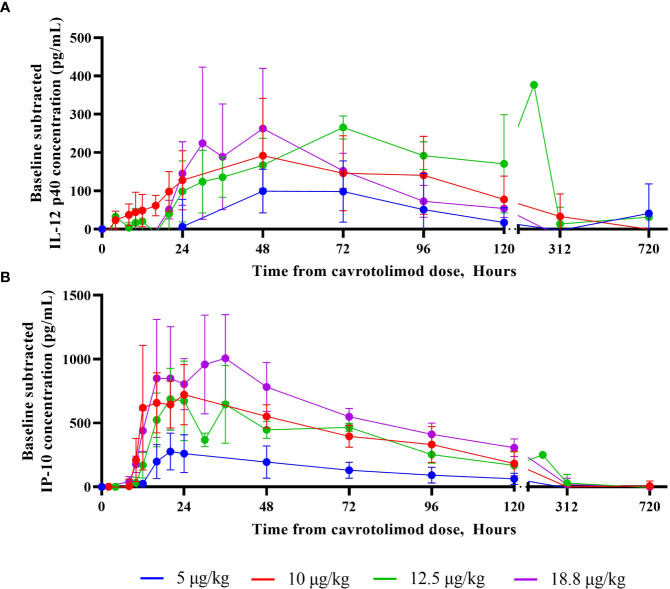
IL-12 p40 and IP-10 cytokine responses as a function of time. A Randox Evidence Investigator or ELISA was used to measure concentrations (pg/mL) of IL-12 p40 and IP-10 in peripheral blood following a single SC dose of cavrotolimod. Each point represents averaged, baseline subtracted values from the four participants in each cohort at the sampling time post-dose of cavrotolimod. **(A)** IL-12 p40. **(B)** IP-10.

For most of the measured cytokines and chemokines, the peak response was observed about 24 hours after cavrotolimod administration, although IL-12 p40 peaked at 48 to 72 hours after dosing (**Figure 4A**). Generally, the IL-6 and IFN-γ concentrations returned to baseline approximately 48 hours after cavrotolimod administration, while IL-1RA, IP-10 (**Figure 4B**), and MCP-1 (at higher doses) required 96 or more hours. Some variability in cytokine response across participants was observed. The non-baseline subtracted peak concentrations of IL-6, IL-12 p40, IL-1RA, IP-10, IFN-γ, and MCP-1 was generally within 2- to 4-fold across participants in one cohort.

**Figure 5:**
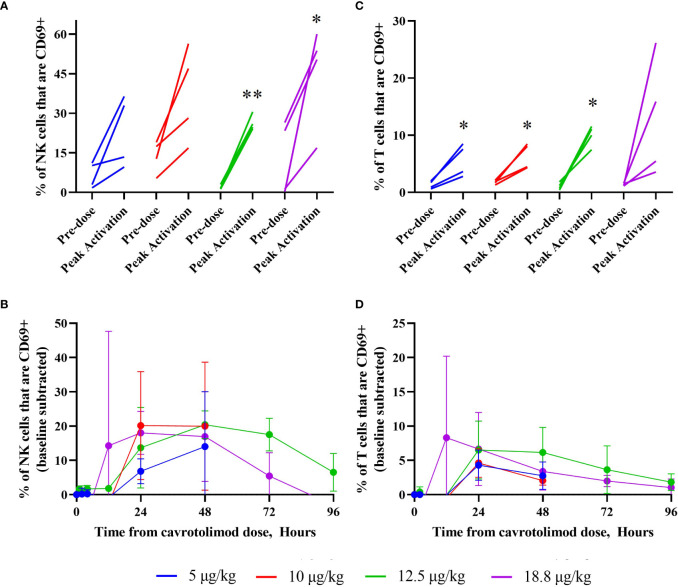
NK and T cell activation as a function of cavrotolimod dose and time. Flow cytometry was used to measure NK and T cell activation in peripheral blood following a single SC dose of cavrotolimod. For graphs **(A, C)**, each line represents a single participant. For each participant, the baseline and peak measurements are indicated, independent of time point when the peak cell activation was observed. For graphs **(B, D)**, each line represents averaged, baseline subtracted values from four participants in a cohort at the sampling time points indicated from receiving the dose of cavrotolimod. **(A, B)** Activated NK cells. **(C, D)** Activated T cells. Paired t-test of comparison of pre- to post-dose values; *p < 0.05; **p < 0.01.

Lymphocyte activation was measured using a flow cytometry-based analysis. The results indicate that cavrotolimod activated NK and T cells in participants following administration. NK cell activation, which is indicated by the presence of the CD69 marker in flow cytometry, was observed in all participants (**Figure 5A**). The NK cell activation peaked at about 48 hours and declined through 96 hours (**Figure 5B**). Mean NK cell activation for the 12.5 µg/kg dose level was significantly increased at 4-, 12-, 48- and 72-hours post-dose compared to the pre-dose levels (p<0.05). T cell activation, which is also measured by the presence of CD69, was also observed in all participants (**Figure 5C**). It generally peaked between 12 and 24 hours, with the activation tapering off through 96 hours (**Figure 5D**). T cell activation in each cohort reached significance (p<0.05) at least once between 24- and 72-hours post-dose compared to pre-dose levels.

No meaningful changes were observed in activated B cells, macrophages or plasmacytoid dendritic cells. For the 5, 10 and 12.5 µg/kg dose levels, 8, 10 and 3-fold increases in the percent of activated monocytes, respectively, were observed at 48 hours. Although these fold changes were relatively large, they were not dose proportional, did not reach statistical significance, and represented only a small fraction (<2%) of the total number of the total monocytes. Further, similar changes were not observed for the 18.8 µg/kg dose level.

**Figure 6:**
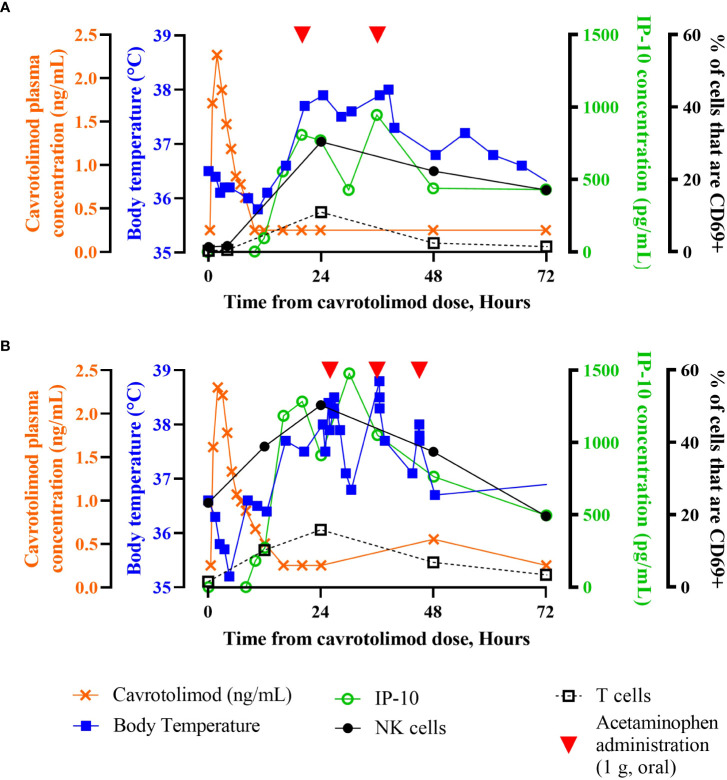
Individual participant time course data. Superimposed PK, PD and body temperature data presented as a function of time from receiving the dose of cavrotolimod for group 3 participant 13012 **(A)** and group 4 participant 14013 **(B)**. Participants 13012 and 14013 received 12.5 µg/kg (0.9 mg) and 18.8 µg/kg (1.1 mg) doses of cavrotolimod, respectively. Each line represents a single data set (PK, PD or body temperature) collected while on study for the respective participant. Administration of 1 g acetaminophen to treat related to AEs are indicated (red arrowheads). IP-10 concentrations and % of cells that are CD69+ are baseline subtracted.

Intraparticipant safety, PK and PD data reveal the kinetics of IP-10 changes, body temperature, cell activation and antipyretic use. **Figure 6** contains individual time course participant data through 72 hours post-dose after either a 12.5 µg/kg (**Figure 6A**) or 18.8 µg/kg (**Figure 6B**) injection of cavrotolimod. For both participants, cavrotolimod was observed in their plasma before meaningful pharmacodynamic effects, followed later by increased concentrations of IP-10. The chemokine release was followed by increases in body temperature (i.e., fever) and NK and T cell activation. Although the plasma concentrations of cavrotolimod peaked earliest and decayed rapidly, the immune cascade elicited by cavrotolimod had a much longer duration, as the activated NK and T cells peaked later with a longer decay to baseline out to 96 hours. When these participants were administered acetaminophen to address fever, in addition to a reduction in body temperature shortly after administration, there were generally corresponding reductions in peripheral chemokine concentrations.

## Discussion

To achieve immune-mediated tumor control, cancer cells must be targeted by immune cells, a process which requires a delicate balance of sufficient neoantigen recognition without widespread autoimmunity. CPIs beneficially promote a shift in that balance toward anti-tumor responses, and do so by blocking certain immune-inhibitory proteins, allowing anti-tumor immune responses to develop or expand. In recent years, the United States Food and Drug Administration (FDA) has approved several CPIs targeting CTLA-4, PD-L1, and PD-1 (38), which have become indispensable treatments for many cancers. However, a considerable number of patients do not respond to or relapse on CPI treatments, a process called antigenic escape (2, 3). Tumor escape from CPI treatment is thought to result from exhausted effector T cells, functionally impaired antigen-presenting cells (APCs), and/or infiltration of tumor-supporting regulatory T cells (T_reg_) and myeloid-derived suppressor cells (MDSCs) (39).

In settings where an anti-tumor response has been initiated but is being repressed, CPI therapies contribute greatly by relieving inhibitory signals. However, CPIs alone cannot promote the initiation of an anti-tumor response, which require immune-stimulating therapies. A rational approach is the use of oligonucleotides containing CpG motifs that can act as activators of TLR9, which is expressed in the endosomes of B and plasmacytoid dendritic cells and induces rapid innate and long-term adaptive immune responses when agonized (5, 8, 11). TLR9 agonists lead to broad activation of immune cells, including APCs and T cells, and suppress T_reg_ and MDSCs in tumor microenvironments (40–42). TLR9-induced immune activation has been extensively characterized in preclinical and clinical studies of cancer (11, 15, 16). TLR9 agonists are often unformulated oligonucleotides. One major challenge associated with use of unformulated oligonucleotides is poor cellular uptake and limited downstream pharmacological activity (43). SNAs are a novel class of therapeutic agents consisting of densely packed and radially oriented oligonucleotides in a 3-dimensional arrangement around a spherical liposomal nanoparticle (23). As a consequence of their 3-D structure (illustrated in **Figure 1**), SNAs have increased cellular uptake, nuclease stability, and affinity to nucleic acid targets (23), characteristics which address many of the challenges associated with unformulated oligonucleotides. Here we described the activity of cavrotolimod, a novel TLR9-agonist SNA that shows potent TLR9 stimulation in a Phase 1 clinical trial.

The primary objective of this study was to assess the safety and tolerability of cavrotolimod following single ascending doses. To meet this objective and to protect the wellbeing of the participants, a CRS and flu-like symptom management strategy was developed and deployed. There is little guidance available in literature about managing CRS risk in healthy participant studies, but it is essential to have a process in place before a trial starts to appropriately classify and manage all CRS symptoms without unnecessarily stopping a trial or its progression. This approach may be useful for many clinical trials of advanced therapies or for approaches that use or release cytokines or chemokines, but we note that the algorithm may require tailoring to the environment and resources available to a particular clinical research unit.

Overall, cavrotolimod was considered safe and well tolerated after single SC injections. The AEs observed in the trial were as expected from the TLR9 agonism-based mechanism of action, nonclinical toxicology studies and from other TLR9 agonist clinical trial results. The absence of a placebo arm in the study did not affect this objective because a small number of placebo participants were unlikely to produce statistically meaningful results. Instead, due to the high inter-participant variability of cytokines, chemokines and other immune markers, the use of intra-participant comparisons of PD markers before and after cavrotolimod administration in the statistical analysis of the safety/tolerability (and PD) was expected to yield more robust results than comparing these effects across placebo and cavrotolimod treated participants.

Cavrotolimod’s mechanism of action and data from other TLR9 agonists showed that cytokine secretion and lymphocyte activation was an expected and desired PD effect. These PD effects would in turn be expected to produce flu-like symptoms including fevers, injection site reactions and lymphadenopathy, which were observed AEs from this Phase 1 study. Injection site reactions and lymphadenopathy were mild and transitory. The lymphocyte and neutrophil count changes observed reflect a well-documented trafficking phenomenon also associated with the pro-inflammatory TLR9 mechanism of action (44). When looking at the overall clinical picture, taking into account AEs, laboratory abnormalities (CRP, neutrophils and lymphocytes), vital signs and lymphadenopathy, the maximum intensity of the flu-like symptoms was mild. Further, no off-target, systemic effects were observed in this study and no other clinically significant abnormalities on safety assessments were observed and thus there was no evidence of any safety signal.

A secondary objective of this study was to assess the PK of cavrotolimod in plasma and urine. C_max_ values were similar for the 5 and 10 µg/kg dose groups whereas dose-proportionate increases were observed from 10 to 18.8 µg/kg; the highest mean C_max_ was 1.8 ng/mL at the highest dose. T_max_ ranged from 2.00 to 2.52 hours over the dose range tested. The sparse plasma concentration values observed are not unusual for a SC administered TLR9 agonist. Importantly, the PK and PD of cavrotolimod are not expected to be temporally related, as was observed in this Phase 1 study and based on the literature. Therefore, similar cytokine and lymphocyte activation measurements are important markers for inclusion in future studies. In future studies utilizing greater doses of cavrotolimod, the PK data may be more robust and informative.

PD analyses of the cytokine release profile and lymphocyte subset activation confirmed the desired effects of cavrotolimod. Cavrotolimod elicited a broad Th1-type immune response in an approximately dose-proportional manner and activated T cells and NK cells. Regarding the cytokine response, cavrotolimod has robust PD activity but did not trigger CRS. IL-12 p40, IL-1RA, IP-10, and MCP-1 were consistently induced across all doses. IP-10 was robustly induced, while IFN-γ and TNF-α were inconsistently induced. The maximum duration of the cytokine PD effect generally appears to be approximately five days. This duration was an important factor underlying the selection of the weekly dosing interval selected for a follow on clinical trial in patients. NK cells are cytotoxic lymphocytes critical to the innate immune system, while T cells play a central role in cell-mediated immunity, so the activation of these cells bodes well for the hypothesis that cavrotolimod will produce anti-tumor immune responses. The NK cell activation peaked at about 48 hours and declined through to 96 hours. T cell activation, which was measured by the presence of CD69, was observed in all participants and generally peaked between 12 and 24 hours, with the activation tapering off through to 96 hours.

The kinetics of cytokine and chemokine expression, and cell activation observed during this study suggest a weekly dosing interval, thus fulfilling the final secondary objective of this study. Indeed, weekly cavrotolimod dosing was used for the first three cycles in a Phase 1b/2 study of cavrotolimod in advanced cancer patients (45). Similar cytokine and lymphocyte activation measurements were used for the Phase 1b study to help inform the selection of the recommended Phase 2 dose.

Cavrotolimod’s PD activity compares favorably to other TLR9 agonists tested in healthy participant studies. PF-03512676, an unformulated TLR9 agonist, did not produce changes in IFNγ or IL-12 cytokine levels after SC administration at a dose of 20 µg/kg (32), while cavrotolimod produced an increase in the plasma concentration of those cytokines at a similar dose of 18.8 µg/kg. IL-12 has been demonstrated to regulate both innate and adaptive immunities in cancer therapy (46), and IFNγ drives PD-L1 expression (47), which correlates to clinical responses to anti-PD-1 and anti-PD-L1 antibody therapy (37). Lefitolimod, another TLR9 agonist previously in a Phase 3 clinical trial, produced a 7-fold increase in cohort mean IP-10 concentrations in participant serum after a 60 mg dose (equivalent to 923 µg/kg for a 65 kg participant) (48). In comparison, cavrotolimod produced a 32-fold increase in IP-10 over baseline at a dose of 18.8 µg/kg. Overall, this Phase 1 study confirmed that cavrotolimod has robust PD activity but did not trigger an immune cascade or CRS.

The PD effects observed in healthy participants in this Phase 1 study suggest that cavrotolimod may synergize effectively with CPI antibodies to initiate and sustain the cancer immunity cycle, which is a seven-step model of how the immune system initiates and propagates anti-tumor immunity (4). Cavrotolimod’s activation of NK cells and T cells, along with induction of TNF, IL-12 and IP-10 cytokines, address at least four of the seven steps in the cycle while CPIs are effective at addressing two other steps. In the context of the cancer immunity cycle and data collected from this Phase 1 study, cavrotolimod has promising PD characteristics for use in combination with CPIs in the first line setting or in patients with disease resistant to PD-1/PD-L1 blockade.

The safety and PD results of this study are compelling support for the use of TLR9-agonist cavrotolimod with CPIs in populations with advanced cancer. On the basis of these results, cavrotolimod was studied in a Phase 1b/2 clinical trial in combination with pembrolizumab or cemiplimab (34, 45) in advanced or metastatic skin cancers.

## Data availability statement

The raw data supporting the conclusions of this article will be made available by the authors, without undue reservation.

## Ethics statement

The studies involving human participants were reviewed and approved by Health Research Authority - London Bridge Research Ethics Committee Skipton House 80 London Road SE1 6LH. The patients/participants provided their written informed consent to participate in this study.

## Author contributions

AB, UL, and WD designed and interpreted the Phase 1 clinical trial study. SM and WD supervised the study as Sponsor, prepared the figures and wrote the manuscript. All authors reviewed the manuscript. All authors contributed to the article and approved the submitted version.

## References

[1] Vilgelm AE Johnson DB Richmond A. Combinatorial approach to cancer immunotherapy: strength in numbers. J Leukoc Biol (2016) 100:275–90. doi: 10.1189/jlb.5RI0116-013RR PMC6608090 27256570

[2] Topalian SL Hodi FS Brahmer JR Gettinger SN Smith DC McDermott DF. Safety, activity, and immune correlates of anti-PD-1 antibody in cancer. N Engl J Med (2012) 366:2443–54. doi: 10.1056/NEJMoa1200690 PMC3544539 22658127

[3] Postow MA Callahan MK Wolchok JD. Immune checkpoint blockade in cancer therapy. J Clin Oncol (2015) 33:1974–82. doi: 10.1200/JCO.2014.59.4358 PMC4980573 25605845

[4] Chen DS Mellman I. Oncology meets immunology: the cancer-immunity cycle. Immunity (2013) 39:1–10. doi: 10.1016/j.immuni.2013.07.012 23890059

[5] Tokunaga T Yamamoto H Shimada S Abe H Fukuda T Fujisawa Y. Antitumor activity of deoxyribonucleic acid fraction from mycobacterium bovis BCG. i. isolation, physicochemical characterization, and antitumor activity. J Natl Cancer Inst (1984) 72:955–62. doi: 10.1093/JNCI/72.4.955 6200641

[6] Sato Y Roman M Tighe H Lee D Corr M Nguyen MD. Immunostimulatory DNA sequences necessary for effective intradermal gene immunization. Science (1996) 273:352–4. doi: 10.1126/science.273.5273.352 8662521

[7] Krieg AM Yi AK Matson S Waldschmidt TJ Bishop GA Teasdale R. CpG motifs in bacterial DNA trigger direct b-cell activation. Nature (1995) 374:546–9. doi: 10.1038/374546a0 7700380

[8] Hemmi H Takeuchi O Kawai T Kaisho T Sato S Sanjo H. A toll-like receptor recognizes bacterial DNA. Nature (2000) 408:740–5. doi: 10.1038/35047123 11130078

[9] Bauer S Kirschning CJ Hacker H Redecke V Hausmann S Akira S. Human TLR9 confers responsiveness to bacterial DNA *via* species-specific CpG motif recognition. Proc Natl Acad Sci USA (2001) 98:9237–42. doi: 10.1073/pnas.161293498 PMC55404 11470918

[10] Klinman DM Yi AK Beaucage SL Conover J Krieg AM. CpG motifs present in bacteria DNA rapidly induce lymphocytes to secrete interleukin 6, interleukin 12, and interferon gamma. Proc Natl Acad Sci USA (1996) 93:2879–83. doi: 10.1073/pnas.93.7.2879 PMC39727 8610135

[11] Krieg AM. Therapeutic potential of toll-like receptor 9 activation. Nat Rev Drug Discovery (2006) 5:471–84. doi: 10.1038/nrd2059 16763660

[12] Vollmer J Weeratna R Payette P Jurk M Schetter C Laucht M. Characterization of three CpG oligodeoxynucleotide classes with distinct immunostimulatory activities. Eur J Immunol (2004) 34:251–62. doi: 10.1002/eji.200324032 14971051

[13] Verthelyi D Ishii KJ Gursel M Takeshita F Klinman DM. Human peripheral blood cells differentially recognize and respond to two distinct CPG motifs. J Immunol (2001) 166:2372–7. doi: 10.4049/jimmunol.166.4.2372 11160295

[14] Marshall JD Fearon K Abbate C Subramanian S Yee P Gregorio J. Identification of a novel CpG DNA class and motif that optimally stimulate b cell and plasmacytoid dendritic cell functions. J Leukoc Biol (2003) 73:781–92. doi: 10.1189/jlb.1202630 12773511

[15] Scheiermann J Klinman DM. Clinical evaluation of CpG oligonucleotides as adjuvants for vaccines targeting infectious diseases and cancer. Vaccine (2014) 32:6377–89. doi: 10.1016/j.vaccine.2014.06.065 PMC4252359 24975812

[16] Krieg AM. CpG still rocks! update on an accidental drug. Nucleic Acid Ther (2012) 22:77–89. doi: 10.1089/nat.2012.0340 22352814

[17] Melisi D Frizziero M Tamburrino A Zanotto M Carbone C Piro G. Toll-like receptor 9 agonists for cancer therapy. Biomedicines (2014) 2:211–28. doi: 10.3390/biomedicines2030211 PMC5344222 28548068

[18] Dimitriou F Hauschild A Mehnert JM Long GV. Double trouble: Immunotherapy doublets in melanoma–approved and novel combinations to optimize treatment in advanced melanoma. In: American Society of clinical oncology educational book, vol. 42 VA: ASCO Publications in Alexandria. (2022). p. 745–66. 10.1200/EDBK_351123 35658500

[19] Jensen SA Day ES Ko CH Hurley LA Luciano JP Kouri FM. Spherical nucleic acid nanoparticle conjugates as an RNAi-based therapy for glioblastoma. Sci Transl Med (2013) 5:209ra152. doi: 10.1126/scitranslmed.3006839 PMC4017940 24174328

[20] Kouri FM Hurley LA Daniel WL Day ES Hua Y Hao L. miR-182 integrates apoptosis, growth, and differentiation programs in glioblastoma. Genes Dev (2015) 29:732–45. doi: 10.1101/gad.257394.114 PMC4387715 25838542

[21] Lewandowski KT Thiede R Guido N Daniel WL Kang R Guerrero-Zayas MI. Topically delivered tumor necrosis factor-alpha-Targeted gene regulation for psoriasis. J Invest Dermatol (2017) 137:2027–30. doi: 10.1016/j.jid.2017.04.027 PMC8405255 28502802

[22] Randeria PS Seeger MA Wang XQ Wilson H Shipp D Mirkin CA. siRNA-based spherical nucleic acids reverse impaired wound healing in diabetic mice by ganglioside GM3 synthase knockdown. Proc Natl Acad Sci USA (2015) 112:5573–8. doi: 10.1073/pnas.1505951112 PMC4426446 25902507

[23] Radovic-Moreno AF Chernyak N Mader CC Nallagatla S Kang RS Hao L. Immunomodulatory spherical nucleic acids. Proc Natl Acad Sci USA (2015) 112:3892–7. doi: 10.1073/pnas.1502850112 PMC4386353 25775582

[24] Mirkin CA Letsinger RL Mucic RC Storhoff JJ. A DNA-based method for rationally assembling nanoparticles into macroscopic materials. Nature (1996) 382:607–9. doi: 10.1038/382607a0 8757129

[25] Cutler JI Auyeung E Mirkin CA. Spherical nucleic acids. J Am Chem Soc (2012) 134:1376–91. doi: 10.1021/ja209351u 22229439

[26] Banga RJ Chernyak N Narayan SP Nguyen ST Mirkin CA. Liposomal spherical nucleic acids. J Am Chem Soc (2014) 136:9866–9. doi: 10.1021/ja504845f PMC4280063 24983505

[27] Barnaby SN Sita TL Petrosko SH Stegh AH Mirkin CA. Therapeutic applications of spherical nucleic acids. Cancer Treat Res (2015) 166:23–50. doi: 10.1007/978-3-319-16555-4_2 25895863

[28] Choi CH Hao L Narayan SP Auyeung E Mirkin CA. Mechanism for the endocytosis of spherical nucleic acid nanoparticle conjugates. Proc Natl Acad Sci USA (2013) 110:7625–30. doi: 10.1073/pnas.1305804110 PMC3651452 23613589

[29] Patel PC Giljohann DA Daniel WL Zheng D Prigodich AE Mirkin CA. Scavenger receptors mediate cellular uptake of polyvalent oligonucleotide-functionalized gold nanoparticles. Bioconjug Chem (2010) 21:2250–6. doi: 10.1021/bc1002423 PMC3241523 21070003

[30] Wu XA Choi CH Zhang C Hao L Mirkin CA. Intracellular fate of spherical nucleic acid nanoparticle conjugates. J Am Chem Soc (2014) 136:7726–33. doi: 10.1021/ja503010a PMC4046773 24841494

[31] Nallagatla S Anderson BR Anantatmula S Kandimalla ER. Spherical nucleic acids targeting toll-like receptor 9 enhance antitumor activity in combination with anti-PD-1 antibody in mouse tumor models. Cancer Res (2017) 77(13_Supplement):4706. doi: 10.1158/1538-7445.AM2017-4706

[32] Krieg AM Efler SM Wittpoth M Al Adhami MJ Davis HL. Induction of systemic TH1-like innate immunity in normal volunteers following subcutaneous but not intravenous administration of CPG 7909, a synthetic b-class CpG oligodeoxynucleotide TLR9 agonist. J Immunother (2004) 27:460–71. doi: 10.1097/00002371-200411000-00006 15534490

[33] Lee DW Gardner R Porter DL Louis CU Ahmed N Jensen M. Current concepts in the diagnosis and management of cytokine release syndrome. Blood (2014) 124:188–95. doi: 10.1182/blood-2014-05-552729 PMC4093680 24876563

[34] Exicure. A phase 1 study of AST 008 in healthy subjects. NCT03086278 (2018). Available at: https://ClinicalTrials.gov/show/NCT03086278.

[35] Rynkiewicz D Rathkopf M Sim I Waytes AT Hopkins RJ Giri L. Marked enhancement of the immune response to BioThrax(R) (Anthrax vaccine adsorbed) by the TLR9 agonist CPG 7909 in healthy volunteers. Vaccine (2011) 29:6313–20. doi: 10.1016/j.vaccine.2011.05.047 21624418

[36] Aryan Z Holgate ST Radzioch D Rezaei N. A new era of targeting the ancient gatekeepers of the immune system: toll-like agonists in the treatment of allergic rhinitis and asthma. Int Arch Allergy Immunol (2014) 164:46–63. doi: 10.1159/000362553 24853609

[37] Patel SP Kurzrock R. PD-L1 expression as a predictive biomarker in cancer immunotherapy. Mol Cancer Ther (2015) 14:847–56. doi: 10.1158/1535-7163.MCT-14-0983 25695955

[38] Hargadon KM Johnson CE Williams CJ. Immune checkpoint blockade therapy for cancer: An overview of FDA-approved immune checkpoint inhibitors. Int Immunopharmacol (2018) 62:29–39. doi: 10.1016/j.intimp.2018.06.001 29990692

[39] Spranger S. Mechanisms of tumor escape in the context of the T-cell-inflamed and the non-t-cell-inflamed tumor microenvironment. Int Immunol (2016) 28:383–91. doi: 10.1093/intimm/dxw014 PMC4986232 26989092

[40] Ashkar AA Rosenthal KL. Toll-like receptor 9, CpG DNA and innate immunity. Curr Mol Med (2002) 2:545–56. doi: 10.2174/1566524023362159 12243247

[41] Murad YM Clay TM. CpG oligodeoxynucleotides as TLR9 agonists: therapeutic applications in cancer. BioDrugs (2009) 23:361–75. doi: 10.2165/11316930-000000000-00000 19894778

[42] Zoglmeier C Bauer H Norenberg D Wedekind G Bittner P Sandholzer N. CpG blocks immunosuppression by myeloid-derived suppressor cells in tumor-bearing mice. Clin Cancer Res (2011) 17:1765–75. doi: 10.1158/1078-0432.CCR-10-2672 21233400

[43] Juliano RL. The delivery of therapeutic oligonucleotides. Nucleic Acids Res (2016) 44:6518–48. doi: 10.1093/nar/gkw236 PMC5001581 27084936

[44] Haining WN Davies J Kanzler H Drury L Brenn T Evans J. CpG oligodeoxynucleotides alter lymphocyte and dendritic cell trafficking in humans. Clin Cancer Res (2008) 14:5626–34. doi: 10.1158/1078-0432.CCR-08-0526 18765557

[45] Exicure. Intratumoral cavrotolimod combined with pembrolizumab in patients with advanced solid tumors. NCT03684785 (2018). Available at: https://clinicaltrials.gov/ct2/show/NCT03684785.

[46] Lu X. Impact of IL-12 in cancer. Curr Cancer Drug Targets (2017) 17:682–97. doi: 10.2174/1568009617666170427102729 28460617

[47] Concha-Benavente F Srivastava RM Trivedi S Lei Y Chandran U Seethala RR. Identification of the cell-intrinsic and -extrinsic pathways downstream of EGFR and IFNgamma that induce PD-L1 expression in head and neck cancer. Cancer Res (2016) 76:1031–43. doi: 10.1158/0008-5472.CAN-15-2001 PMC4775348 26676749

[48] Manuel S Kerstin K Detlef O Matthias S Burghardt W Alfredo Z. The immunotherapeutic TLR-9 agonist MGN1703 – pharmacokinetic and pharmacodynamic data from healthy volunteers and cancer patients. Eur J Cancer (2015) 51:S12. doi: 10.1016/j.ejca.2015.01.049

