# Supplementary material for: A first-in-human phase 1 study of cavrotolimod, a TLR9 agonist spherical nucleic acid, in healthy participants: Evidence of immune activation
Source: Front Immunol. 2022 Dec 13;13:1073777. doi: 10.3389/fimmu.2022.1073777 (PMC9792500; doi:10.3389/fimmu.2022.1073777)
Supplement: Supplementary file 1 [file DataSheet_1.pdf]

Supplementary Material

1 Materials and Methods

1.1 Pharmacokinetic Assessments

Venous blood samples were collected to assess the concentration cavrotolimod in plasma (K_2EDTA). Blood was collected pre- and post-dose through Day 6. Urine samples for cavrotolimod concentration measurements were taken from the total urine sample provided pre-dose and post-dose through Day 2.

Cavrotolimod concentrations were assessed in plasma with a validated nucleic acid sandwich assay, in which the oligonucleotide was detected by first digesting the sample in lysis buffer using a sonicator and proteinase K treatment. Then, the analyte was annealed through Watson-Crick base pairing to a complementary template probe which was biotinylated at the 5'-end of the sequence. Next, the hybrid complex was immobilized by plating on a streptavidin-coated plate. Then, a ligation detection probe with digoxigenin at the 5'-end was added to hybridize with the nine-nucleotide overhang of the template probe. A ligation reaction to ligate the analyte and ligation detection probe occurred with T4 polynucleotide kinase and the T4 DNA ligase. Unligated probe was subsequently removed by washing and S1 nuclease. Finally, a detection step was carried out with anti-digoxigenin-sulfo-tag (ruthenium) and electrochemiluminescence (ECL) intensity was measured using an MSD Sector S 600. The signal achieved in the samples was compared to a standard curve to measure the amount of cavrotolimod in the sample. The quantification range of the assay is 0.5000 to 150.0 ng cavrotolimod oligonucleotide/mL in human plasma (K_2EDTA). The sample analysis was performed by Algorithme Pharma (Laval Quebec, Canada). Calculation of the pharmacokinetic parameters was performed by Certara Strategic Consulting (Montreal, Quebec, Canada).

Urine concentrations of cavrotolimod were assessed with a validated liquid chromatography method and fluorescence-based detection by annealing the analyte to a complementary, fluorescently labeled peptide nucleic acid (PNA) probe through Watson-Crick base pairing. The samples were first digested with proteinase K at 65 °C in the presence of a lysis buffer, followed by sonication. The samples were then extracted with dichloromethane. The digested samples were heated to 95 °C in the presence of the PNA probe and then rapidly cooled to 20 °C to form duplexes of the PNA probe and cavrotolimod oligonucleotide. Ion exchange HPLC was used to separate the duplexes and the excess single-stranded PNA probe. A fluorescence detector was used to detect the annealed probe for the indirect quantification of cavrotolimod. The signal achieved in the samples was compared to a standard curve to measure the amount of cavrotolimod in the sample. The quantification range of the assay is 10.0 to 1000 ng cavrotolimod oligonucleotide/mL in human urine. The sample analysis was performed by Covance Laboratories, Inc. (Salt Lake City, UT, USA).

Non-compartmental analysis was used for estimation of PK parameters. Area under the curve from 0 to 24 hours after the dose ($AUC_{0\text{-}24hr}$) and from 0 hours to the last sampling time point ($AUC_{0\text{-}tlast}$) were calculated using the linear/log trapezoidal method, applying the linear trapezoidal rule up to the maximum plasma concentration (C_{max}) and the log trapezoidal rule for the remainder of the curve. The actual time points for blood and urine sampling were used in the analysis. Samples below limit of quantitation (LOQ) prior to the first quantifiable concentration were set to zero. Samples with

concentrations below LOQ in the terminal phase (after last quantifiable concentration) were omitted from the analysis. Other PK parameters were calculated according to standard equations.
